# Optimization of Alcian blue pH 1.0 histo-staining protocols to match mass spectrometric quantification of sulfomucins and circumvent false positive results due to sialomucins

**DOI:** 10.1093/glycob/cwab091

**Published:** 2021-08-18

**Authors:** János Tamás Padra, Sara K Lindén

**Affiliations:** Department of Medical Chemistry and Cell Biology, University of Gothenburg, Box 440, 40530, Gothenburg, Sweden; Department of Medical Chemistry and Cell Biology, University of Gothenburg, Box 440, 40530, Gothenburg, Sweden

**Keywords:** Alcian blue, mass spectrometry, mucin, O-glycosylation, sulfation

## Abstract

Sulfomucins are in some body locations and species a normal occurrence, whereas in other situations, are a sign of pathology. Sulfomucin content on histological sections and isolated material is frequently analyzed with Alcian blue staining at pH 1.0. However, since the stain detects the charge, a high density of other charged molecules, such as sialic acids, has potential to impede specificity. Here, we compared the outcome from four staining protocols with the level of sulfation determined by liquid chromatography–tandem mass spectrometric analysis on samples from various tissues with variable sulfation and sialylation levels. We found that a protocol we designed, including rinsing with MetOH and 0.5 M NaCl buffer at pH 1.0, eliminates the false positive staining of tissues outperforming commonly recommended solutions. In tissues with low-to-moderately sulfated mucins (e.g. human stomach and salmonid epithelia), this method enables accurate relative quantification (e.g. sulfate scoring comparisons between healthy and diseased tissues), whereas the range of the method is not suitable for comparisons between tissues with high sulfomucin content (e.g. pig stomach and colon).

## Introduction

Goblet cells are a dominant feature of many epithelia, and they continuously secrete mucus, which is mainly composed of heavily glycosylated mucins (Quintana-Hayashi et al. 2018). Sialylation and sulfation are terminal modifications of mucin *O*-glycans that differ between species, individuals, epithelia within each individual and health status as determined by mass spectrometry (MS) and are summarized in Supplementary Table SI (Padra et al. 2018; Quintana-Hayashi et al. 2018; Benktander et al. 2019). For example, Atlantic salmon mucin glycan structures are highly sialylated (Jin et al. 2015; Benktander et al. 2020), while human and porcine gastric mucins have comparatively low sialylation (Jin et al. 2017; Padra et al. 2018). Mucin glycosylation changes (Linden S et al. 2008; Linden SK et al. 2008; Venkatakrishnan et al. 2017) are important, can affect host–pathogen interactions and can serve as biomarkers and diagnostic tools (Linden S et al. 2008; McGuckin et al. 2011; Benktander et al. 2021). Sulfomucins’ detection in early diagnosis plays an important role in determining patient treatment (Mishra et al. 2020), e.g. in determining the stage of gastrointestinal pathologies (Filipe 1969; Filipe 1972; Rugge et al. 2011). However, in addition to human diagnostics, studies often involve animals and tissues where the sulfation and sialylation levels are unknown. Different protocols for staining of sulfomucins are used, including Alcian blue (AB) pH 1.0 staining (Cohen et al. 2000) and colloidal iron/high iron diamine (HID) stains (Spicer 1965). These stains have the potential to negatively impact staining specificity by the nonspecific staining of sialic acids (Reid et al. 1989). Other methods like Alcian yellow (AY) pH 1.0 or the laborious and the often incomplete, digestion of sialic acids before AB2.5 staining are also error-prone (Sorvari and Nanto 1971; Scillitani et al. 2012; Padra et al. 2014). Due to ease of use, AB pH 1.0 has gained popularity compared to the more traditional iron stains (Samar et al. 1999; Cohen et al. 2000). Upon trying one of the widely used AB pH 1.0 protocols on Atlantic salmon histology sections, we noticed a large discrepancy between the histological staining of the mucin-producing goblet cells and results obtained by MS.

The main objectives of this study were therefore to compare the results obtained by several AB staining protocols and to identify the protocol(s) that best reflects the sulfation abundance results obtained by MS in the presence of different sialic acid levels.

## Results

### AB staining of Polyvinylidene Fluoride (PVDF) membrane-blotted mucins is a good indicator of sialylation and sulfation levels but has positive error in the lower range of detection

First, we tested the AB2.5 and AB1.0 protocols for specificity on mucins with variable sialylation and sulfation dotted onto PVDF membranes. Both the AB2.5 and AB1.0 staining methods correlated well with the total sialylation and sulfation versus the sulfation level, determined by MS, but yielded unspecific weak staining in negative samples (detailed description in the Supplementary file and Supplementary Figure S1A–C).

### Rinsing tissue sections with 0.1 M HCl/MetOh 9:1 (pH 1.0) followed by 0.5 M NaCl/HCl (pH 1.0) reduces false positive sulfate staining

**Fig. 1:**
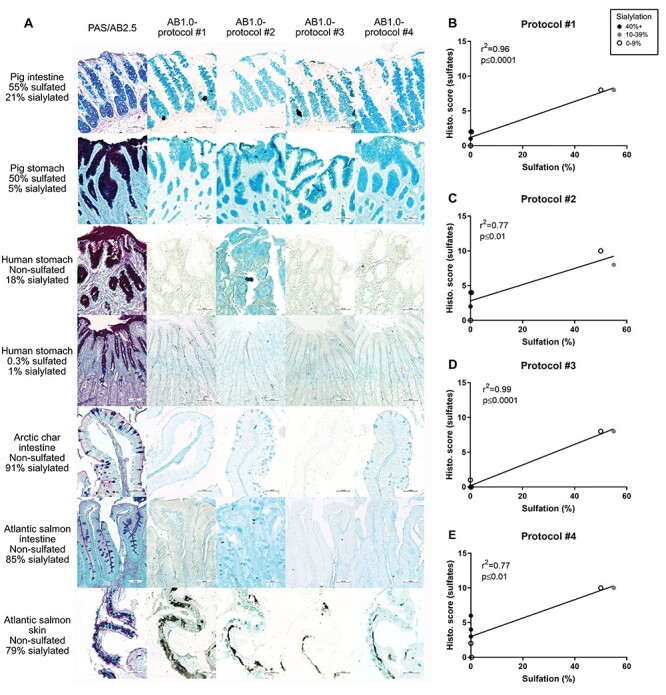
PAS/AB2.5 and four varieties of AB1.0 staining to visualize sulfation in animal tissues and correlation with relative abundance of sulfate. (**A**) Tissues with decreasing levels of sulfation (quantified by MS), from top to bottom, were stained. PAS/AB2.5 shows neutral mucins with magenta color and acidic mucins with blue color. The black in the Atlantic salmon skin samples is an artifact present in skin with this stain, but goblet cells can be observed in areas without black. 100× magnification. (**B**–**E**) Sulfate levels of the seven tissues studied correlated positively with all four protocols. Protocol #3 (**D**) shows the strongest association between the parameters (*r*^2^ = 0.99, *P* ≤ 0.0001, *n* = 7), followed by protocol #1 (**B**) (*r*^2^ = 0.96, *P* ≤ 0.01, *n* = 7), protocol #4 (**E**) (*r*^2^ = 0.77, *P* ≤ 0.01, *n* = 7) and protocol #2 (**C**) (*r*^2^ = 0.77, *P* ≤ 0.01, *n* = 7). Samples having high relative abundance of sialic acids (black dots) cause elevated staining levels with protocols #1, #2 and #4 but not with #3. Sialic acid abundance is marked with black dots over 40%, gray dots between 10% and 39% and white dots at or below 9%. Statistics: Pearson’s correlation test (*r*^2^, *n* = 7).

Mucosal tissue sections (*n* = 7) were stained using four different AB pH 1.0 staining protocols (Supplementary Table SII) to visualize sulfation and with a standard Periodic Acid Schiff (PAS)/AB pH 2.5 staining protocol to visualize sialic acids, sulfates and neutral mucins (Figure 1A). Figure 1A shows representative images of tissues stained with each of the protocols. With all four AB pH 1.0 protocols, there was a clearly stronger staining of porcine tissues (55% and 50% relative abundance of sulfation as determined by MS) than that of the human (0% and 0.3% sulfation) and fish tissues (sulfation not detected).

Results of the microscopy scoring (scale 0–10) were correlated with the sulfation levels measured with MS (Figure 1B–E, *n* = 7). Protocol #3 was found to correlate best with the sulfate levels quantified with MS (Figure 1D). The other protocols (#1, #2 and #4) had a weaker correlation due to that highly sialylated samples scored positive in spite of lacking sulfation (Figure 1B, C and E).

### Rinsing tissue sections with MetOh/HCl (pH 1.0) followed by 0.5 M NaCl/HCl (pH 1.0) (“protocol #3”) yields accurate relative quantification of sulfates in tissues with <24% sulfation

**Fig. 2:**
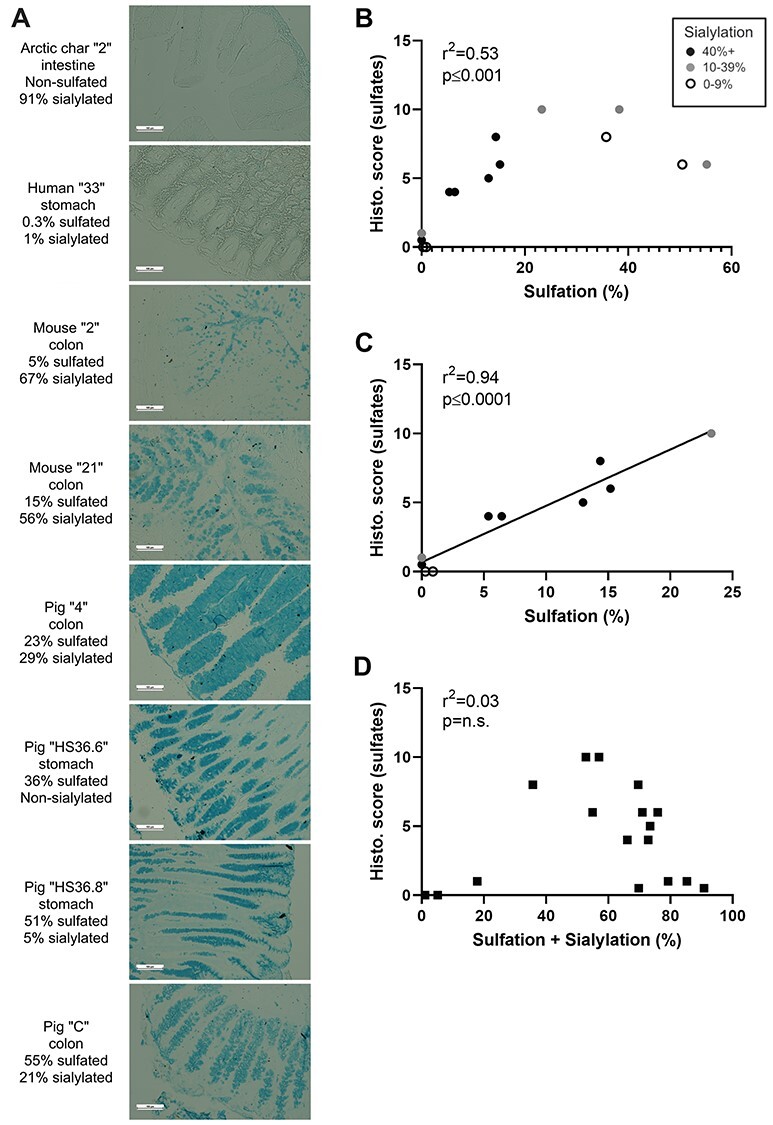
Relationship between MS quantification versus staining with “protocol #3” for sulfates. (**A**) Eight representative tissues with increasing levels of sulfation (quantified by MS) from top to bottom were stained with “protocol #3.” Arctic char: There is weak staining in the nonmucosal region and no apparent staining in the mucosal region. Human stomach: There is no visible staining of the tissue. Murine colons: There is moderate staining in both the 5% sulfated and the 15% sulfated mucus in the goblet cells and the in the luminal space. Pig tissues: All pig tissues stained strong for sulfates. Out of the four tissues, the stomach samples (36% and 51% sulfated) show slightly stronger staining than the colon sections (23% and 55% sulfated). 100× magnification. (**B**) Correlation analysis showed positive correlation between the two quantification methods (*r*^2^ = 0.73, *P* ≤ 0.001, *n* = 17), however, the linear relationship disappeared at sulfation values >24%. (**C**) There is a strong correlation between histological staining and MS quantification for samples with low-to-moderate sulfation (<25% abundance; *r*^2^ = 0.97, *P* ≤ 0.0001, *n* = 13). On graphs (**B**) and (**C**), the samples with high sialic acid content (black dots) have histological scores in line with the samples with low sialic acid content (white dots). (**D**) The staining scores of “protocol #3” did not correlate with the total abundance of sialic acids and sulfates in the samples (*r*^2^ = 0.03, *P* = n.s., *n* = 17). Sialic acid abundance is marked with black dots over 40%, gray dots between 10% and 39% and white dots at or below 9%. Statistics: Pearson’s correlation test (*r*^2^).

To test protocol #3 further, we stained 17 tissue sections from fish, mice, pig and human with sulfated mucin glycan content ranging from 0% to 55% and with variable sialic acid content. The gradient of blue staining matched the MS results (Figure 2A). Tissues ranging from 0% to 23% sulfation were visibly distinguishable from each other based on staining intensity, and neither Arctic char intestine (0% sulfation) nor human stomach (0.3% sulfation) were positive (Figure 2A). The porcine gastric and colon tissues ranging from 23% to 55% sulfation all had stronger blue staining than the 15% sulfated mouse colon tissue (Figure 2A). However, the 51% and 55% sulfated porcine tissues did not stain stronger than the 23% and 36% sulfated porcine tissues (Figure 2A).

We found a positive correlation between the histological scoring of protocol #3 and the sulfation levels quantified by MS analysis (Figure 2B, *r*^2^ = 0.53, *P* ≤ 0.001, *n* = 17). The shape of the curve was only linear until ca. 25% sulfation score, which is due to the variable staining strengths and a proportionally weaker staining of the porcine tissues with the higher sulfation levels (Figure 2A). Correlating the MS and AB stain results, excluding the high sulfate porcine samples, resulted in a very strong linear correlation (Figure 2C, *r*^2^ = 0.94, *P* ≤ 0.0001, *n* = 13). The staining score did not correlate with the sum of the sialylation and sulfation (Figure 2D; *r*^2^ = 0.03, *P* = n.s., *n* = 17). This indicates that unspecific AB staining of sialic acids is not likely with this protocol.

## Discussion

Here, we identified that the commonly used staining protocols with AB at pH 1.0, used for identifying sulfomucins, gave false positive results when sialic acids are present, and therefore, we developed a protocol with more stringent washing steps to avoid this problem. We did not see marked differences in the staining due to the high sialic acid levels on mucins dotted to PVDF membranes. This suggests that the washing steps we applied here, 0.1 M HCl followed by further destaining with methanol three times, prevented the false positive staining of sialomucins. Despite the thorough washing in methanol that dissolves unbound AB particles (Schenk 1981), there seems to be certain physicochemical properties of the mucins that contribute to the appearance of the weak residual stain in the absence of sulfate and sialic acid.

Tissue staining scores of four AB pH 1.0 staining methods differing in the washing steps each resulted in strong positive correlation with the relative abundance of sulfates. However, samples lacking sulfation also bound the blue stain weakly. The most precise method was protocol #3, which did not show a staining level distinguishable from the background staining for the samples with nondetectable sulfate levels using MS. This is in line with the PVDF membrane staining. The similarity between the two methods is the low pH (pH 1.0) and the washing with methanol, which seems to be protective against the undesirable sialomucin staining.

In samples with sulfation levels between 0% and 55%, the correlation between histological scoring of tissues stained with protocol #3 and MS detection is strong but not linear. In the range of 0% to ca. 24% relative abundance of sulfates, the staining intensity follows the sulfation levels closely but reaches a plateau >24%. This means that protocol #3 is a useful tool in the relative quantification of sulfomucins in all samples with low-to-moderate sulfomucin content but only as a qualitative detection method in samples with high sulfation. In this context, we also want to highlight that sulfates ionize efficiently and therefore are most likely somewhat overestimated in the MS data. The 24% should therefore not be used as an absolute value but as an indication for what type of samples are suitable to perform semi-quantification on. The lack of correlation between the summarized relative abundance of sulfation and sialylation on these mucin samples and the histological scores of sulfomucins suggests that the effect of sialic acids is negligible with protocol #3.

Contrary to AB pH 1.0 kit suppliers’ instructions, we have shown that as low as 5% relative abundance of sulfation is detectable with this protocol, as it stains distinctively stronger than tissues having no sulfomucins. Moreover, rinsing with 0.1 M HCl or blotting with paper alone is not enough to avoid false positive staining as we have seen strong staining with some of the nonsulfated samples with protocols #1 and #2. Five out of 10 reviewed commercially available kits suggested washing with 3% acetic acid or running tap water, which is alarming since it has been long established that the pH of the rinsing solution should be kept low and water should be avoided (Lev and Spicer 1964).

In conclusion, we bring attention to that widely used AB pH 1.0 staining kits and protocols lack vital, or provide false, information for accurate assessment of sulfomucins. As a substitute protocol, we recommend using AB pH 1.0 staining followed by three rinses of MetOh/HCl (9:1 v/v; pH 1.0) and three rinses of 0.5 M NaCl/HCl (pH 1.0). This method safely circumvents false positive sialomucin staining, enabling reliable use of the AB pH 1.0 staining method in histopathological applications.

## Materials and methods

The materials and methods used in this study can be found in the supplementary files.

## Supplementary Material

Supplementary_cwab091

## Data Availability

The data underlying this article will be shared on reasonable request to the corresponding author.
