# Supplementary material for: Optimization of Alcian blue pH 1.0 histo-staining protocols to match mass spectrometric quantification of sulfomucins and circumvent false positive results due to sialomucins
Source: Glycobiology. 2021 Aug 18;32(1):6–10. doi: 10.1093/glycob/cwab091 (PMC8881734; doi:10.1093/glycob/cwab091)
Supplement: Supplementary_cwab091 [file supplementary_cwab091.docx]

**Supplementary content:**

Supplementary Table I: page 2-3

Supplementary Table II: page 4

Supplementary results: page 5-6

Supplementary Figure S1: page 6-7

Materials and methods: page 7-12

References: page 12-13

| Species and Organ (n) [Ref] | Sialic acids [%; Median (Range)] | Sulfation [%, Median (Range)] |
| --- | --- | --- |
| Human stomach-healthy (5) (Jin, C., Kenny, D.T., et al. 2017) | 14.9 (15.1) | 0 (0.9) |
| Human stomach-*H. pylori infected* (3) (Jin, C., Kenny, D.T., et al. 2017) | 2.4 (7.0) | 0 (0.3) |
| Human stomach-cancer (4) (Jin, C., Kenny, D.T., et al. 2017) | 22.3 (49.5) | 11.1 (19.5) |
| Porcine stomach-healthy (3) (Padra, M., Adamczyk, B., et al. 2018) | 5.2 (5.9) | 28.1 (21.4) |
| Porcine stomach-*H. suis* infected (4) (Padra, M., Adamczyk, B., et al. 2018) | 4.9 (7.5) | 36.9 (30.8) |
| Porcine colon-healthy (4) (Venkatakrishnan, V., Quintana-Hayashi, M.P., et al. 2017) | 16.6 (7.9) | 53.6 (4.4) |
| Porcine colon-*B. hyodysenteriae* infected (5) (Venkatakrishnan, V., Quintana-Hayashi, M.P., et al. 2017) | 23.6 (11.0) | 36.3 (21.7) |
| Atlantic salmon skin -healthy (8) (Benktander, J., Venkatakrishnan, V., et al. 2019, Jin, C., Padra, J.T., et al. 2015) | 78.5 (18.0) | ≤0.1 |
| Atlantic salmon intestine -healthy (8) (Benktander, J., Venkatakrishnan, V., et al. 2019, Jin, C., Padra, J.T., et al. 2015) | 90.4 (35.9) | ≤0.1 |
| Arctic char intestine -standard fed (5) (Venkatakrishnan, V., Padra, J.T., et al. 2019) | 85.9 (6.4) | ≤0.1 |
| Arctic char intestine -SBM diet (4) (Venkatakrishnan, V., Padra, J.T., et al. 2019) | 84.8 (5.4) | ≤0.1 |

**Supplementary Table I.** **Mass Spectrometry based relative quantification of sialylation and sulfation of mucins from different animals, health status and organs** The table shows the median and range values of the relative abundance of total sialic acids (NeuAc, NeuGc, and KDn) and sulfation (%) as determined by Mass spectrometric analysis. Abbreviations: n = number of individuals; standard fed = fed with fish feed commonly used in aquaculture; SBM diet = fed with meal with added full-fat ground soybean (to induce inflammation).

| Step nr. | **Protocol #1** | **Protocol #2 - Spicer et al., 1965** | **Protocol #3** | **Protocol #4** | **PAS-AB staining** |
| --- | --- | --- | --- | --- | --- |
| 1 | Dewaxing | Dewaxing | Dewaxing | Dewaxing | Dewaxing |
| 2 | Ethanol 99,5 % - 10´ | Ethanol 99,5 % - 10´ | Ethanol 99,5 % - 10´ | Ethanol 99,5 % - 10´ | Ethanol 99,5 % - 10´ |
| 3 | Running tap water - 10´ | Running tap water - 10´ | Running tap water - 10´ | Running tap water - 10´ | Running tap water - 10´ |
| 4 | 0.1M HCl (pH1.0) - 2´ | 0.1M HCl (pH1.0) - 2´ | 0.1M HCl (pH1.0) - 2´ | 0.1M HCl (pH1.0) - 2´ | 3% Acetic acid - 2´ |
| 5 | 1% Alcian blue (pH 1.0) - 30´ | 1% Alcian blue (pH 1.0) - 30´ | 1% Alcian blue (pH 1.0) - 30´ | 1% Alcian blue (pH 1.0) - 30´ | 1% Alcian blue (pH 2.5) - 2h 30´ |
| 6 | Rinse 3 × with 0.1M HCl (pH1.0) |  |  |  |  |
| 7 |  |  | Rinse 3 × with MetOH/0.1M HCl |  |  |
| 8 |  |  | Rinse 3 × with 0.5M NaCl/0.1M HCl (pH1.0) | Rinse 3 × with 0.5M NaCl/ 0.1M HCl (pH1.0) |  |
| 9 |  |  |  |  | 3 % Acetic acid - 1´ then rinse in running tap water - 9´ |
| 10 |  |  |  |  | 1 % Periodic acid - 10´ |
| 11 |  |  |  |  | Running tap water - 5´ |
| 12 |  |  |  |  | Schiff reagent - 15´ |
| 13 |  |  |  |  | Running tap water - 5´ |
| 14 |  |  |  |  | 3 × 0.5 % Sodium meta-bisulfite - 1´ |
| 15 |  |  |  |  | Running tap water - 5´ |
| 16 |  | Blotting with paper |  | Blotting with paper |  |
| 17 | Dehydration | Dehydration | Dehydration | Dehydration | Dehydration |
| 18 | Mounting | Mounting | Mounting | Mounting | Mounting |

**Supplementary Table II. Alcian blue staining protocols used in this study**

**Supplementary results:**

**Alcian blue staining of PVDF membrane-blotted mucins is a good indicator of sialylation and sulfation levels but has positive error in the lower range of detection**

We stained PVDF membrane bound mucins with Periodic acid-Schiff staining (PAS) to visualize neutral mucins. The PAS stain intensity of human healthy gastric mucins was high, porcine stomach and colon mucins intermediate, while human gastric cancer mucins and Arctic char intestinal mucins was low (Supplementary Fig S1A), reflecting the proportion of neutral mucins among these samples. AB pH 2.5 staining (stains both sialic acids and sulfates) was strong in mucins from Arctic char intestine, intermediate in mucins from porcine colon and porcine stomach and low in mucins from human healthy or cancerous stomachs (extracted from the tumor tissue) (Supplementary Fig S1A). AB pH 1.0 staining for sulfate resulted in intermediate intensity in the staining of mucins from porcine stomach and colon and low staining in mucins from human healthy stomach, human gastric cancer and Arctic char intestine.

AB pH 2.5 staining is expected to stain both sialic acids and sulfates, therefore we correlated staining intensity with the sum of the MS abundance of these two groups of glycans in the samples used for the blot (Supplementary Fig S1B). The MS results for pooled sialylation and sulfation and the AB stain at 2.5 correlated well (r=0.93; p≤0.01; n=5; Supplementary Fig S1B). However, the staining intensity was similar, +20% and +22% higher signal compared to background staining, for samples having 9% and 0% of for total abundance of sialic acids and sulfates. The staining method thus can result in false positives in the lower range and low accuracy for samples with low concentration of these structures.

Similarly, AB pH 1.0 stained samples correlated well with sulfation abundance (r=0.96; p≤0.01; n=5; Supplementary Fig 1C). However, the three non-sulfated samples had staining stronger than that of the background (+3.4%, +4.9% and +5.5% difference), suggesting unspecific binding, possibly due to a high level of sialylation present in these samples.

**
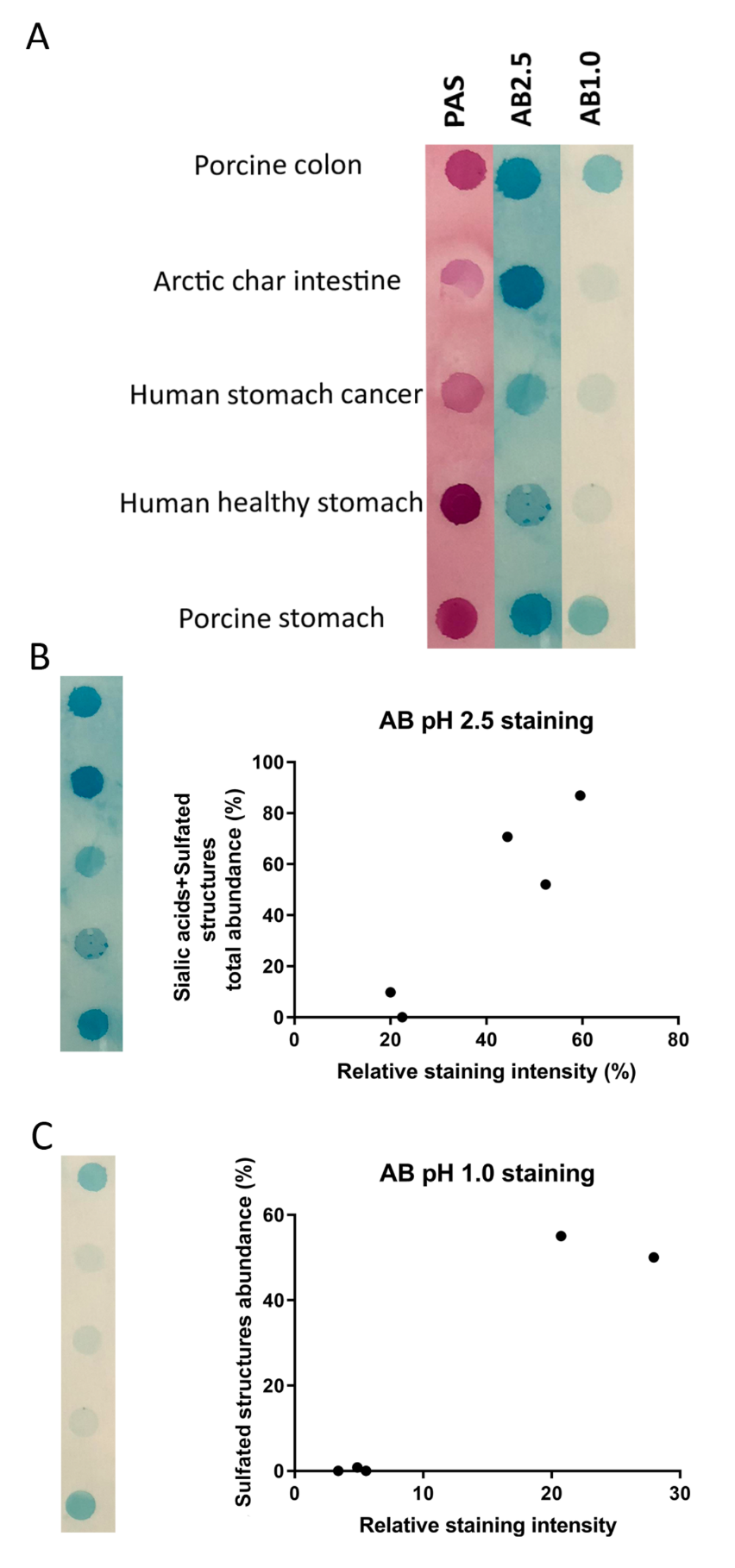
**

**Supplementary Figure S1. PAS/AB1.0 and AB2.5 staining of PVDF blotted mucin samples and their correlation with Mass Spectrometric quantification.** A. Dot-blotted mucin samples to PVDF membrane stained for neutral mucins (PAS), acidic mucins (AB pH2.5) and sulfates (AB pH1.0) using standard staining technique. B. Relative staining intensity (relative to background staining) of the AB pH2.5 staining correlates positively with the total abundance of sialylated and sulfated structures (r=0.93; p≤0.01; n=5). C. Relative staining intensity (relative to background staining) of the AB pH1.0 staining correlates positively with the total abundance of sulfated structures (r=0.96; p≤0.01; n=5).

**Materials and methods:**

**Samples**

Mucus and tissue samples from all animal species and individuals were obtained from adjacent tissues within the same regions to ensure there were no differences in glycosylation of the mucins. Tissue samples were immediately placed in Carnoy´s fixative solution (60% ethanol/ 30% chloroform/ 10% glacial acetic acid). Mucus and tissues for mucus extraction were placed in sampling buffer (10 mM sodium di-hydrogen phosphate, 0.1 mM phenylmethanesulfonyl fluoride (PMSF), pH 6.5)and further processed as described in the next subsection.

Arctic char and Atlantic salmon were sedated in water using metomidate (12.5 mg/l) and killed with a blow to the head. The fish were opened longitudinally and the intestine and a 1 cm^2^ piece of the skin next to the dorsal fin were quickly dissected out. The mucus and mucosa were scraped off using microscopy slides. The experiments on Arctic char and Atlantic salmon were approved by the Ethical Committee for Animal Experimentation in Umeå, Sweden under licence #A62-10 and the Ethical Committee for Animal Experimentation in Gothenburg, Sweden under licence #46/2009, repectively.

Human gastric samples from obese patients were obtained in conjunction with vertical sleeve gastrectomy and collected as described previously (Padra, M., Adamczyk, B., et al. 2019), after written informed consent (Ersta Hospital, Sweden). The study was approved by the Gothenburg ethics board (Dnr 753-14).

Porcine gastric tissues were collected from *Helicobacter suis* inoculated and sham inoculated pigs as described earlier (Padra, M., Adamczyk, B., et al. 2019). Briefly, early-weaned piglets (Piétrain boar × Rattlerow Seghers sow, EC approval number EC2012/129) were (at 4 weeks of age) orally inoculated with 5 mL of pH 5.0 Brucella broth (Becton Dickinson, Erembodegem, Belgium), containing 5 × 10^8^ viable *H. suis* strain HS1 per mL. The control group was given 5 mL of sterile Brucella broth. All pigs were euthanized after 4 weeks.

Porcine colon samples were collected from *Brachyspira hyodysenteriae* inoculated and sham inoculated pigs as described previously (Quintana-Hayashi, M.P., Mahu, M., et al. 2015). Briefly, nine 6 - week old pigs (Piétrain × Danish Large White) received oral inoculation with brain heart infusion broth containing 10^8^ CFU of *B. hyodysenteriae* strain 8dII, or received sterile BHI. After 40 days, the pigs were sacrificed. Midsection samples of the spiral colon were collected for histology and mucin isolation.

Murine distal colon samples were collected from 8-12 week old male wild type (wt) C57BL/6 and C57BL/6 Formyl Peptide Receptor 2 Knock-out (Fpr2^-/-^) mice infected with *Citrobacter rodentium* strain ICC169, as previously described (Sharba, S., Venkatakrishnan, V., et al. 2019). The mice were sacrificed 6 days after inoculation. Distal colon samples of three mice of each genotype were collected for mass spectrometry and histology (Sharba, S., Venkatakrishnan, V., et al. 2019).

**Isolation and purification of mucins.** The pulverized tissues and scraped off crude mucus in sampling buffer were placed into five sample volumes of extraction buffer (6 M GuHCl, 5 mM EDTA, 10 mM sodium phosphate buffer, 0.1 mM PMSF, pH 6.5), dispersed with four strokes with a loose pestle in a Dounce homogenizer and stirred slowly overnight at 4°C. The material was centrifuged at 23,000 × g for 50 min at 4°C (Beckman JA-30 rotor) and the pellet was re-extracted twice with 10 ml extraction buffer. The supernatants from these three extractions were pooled and filled up to 26 ml with extraction buffer. CsCl was slowly stirred into the samples and the samples then transferred to Quick Seal ultracentrifuge tubes (Beckman Coulter). The tubes were filled with 10 mM NaH_2_PO_4_ to give a starting density between 1.35 and 1.39 g/ml (depending on the studied species) and samples were subjected to density gradient centrifugation at 40,000 × g for 90h at 15°C. The fractions were collected from the bottom of the tubes. Density measurements on the fractions were performed using a 300 µl Carlsberg pipette as a pycnometer. DNA content was measured by UV light absorbance at 260 nm, and mucin containing fractions were pooled as to not contain DNA containing fractions. Density gradient fractions were analyzed for carbohydrates as periodate-oxidizable structures in a microtiter-based assay and the mucin containing fractions pooled based on a peak of a high glycan content combined with being of typical mucin density (1.35-1.45 g/mL), as previously described (Padra, J.T., Sundh, H., et al. 2017).

**Liquid-chromatography-tandem mass spectrometry (LC-MS/MS).** Approximately 100 µg of mucins, dot blotted on PVDF membrane (Millipore), stained with Alcian blue 8GX solution in acetic acid, were excised and subjected to reductive *β*-elimination (0.5 M sodium borohydride in 50 mM sodium hydroxide, 16 h at 50 °C) to release the *O*-glycans. The reaction was quenched by glacial acetic acid and the material desalted using a cation exchange resin packed on top of a C18 ziptip column (Millipore). Excess borate was extracted as methyl esters by repeated evaporation. Released *O*-glycans were analyzed by LC-MS/MS as previously described (Padra, M., Adamczyk, B., et al. 2019), using a 10 cm × 250 µm column, containing 5 µm porous graphitized carbon (PGC) particles (Thermo Scientific, Waltham, MA, USA) connected to an LTQ mass spectrometer (Thermo Scientific). *O*-glycans were eluted at a flow rate of 250 nl/min using a linear gradient from 0 to 40% acetronitrile in 10 mM ammonium bicarbonate . Electrospray ionization-mass spectrometry (ESI-MS) was performed in negative ion polarity with an electrospray voltage of 3.5 kV, capillary voltage of -33.0 V, and capillary temperature of 300°C. The following scan events were used: MS full scan (*m/z* 380-2000) and data-dependent tandem MS (MS/MS) scans after collision-induced dissociation (CID) on precursor ions at a normalized collisional energy of 35% with a minimum signal of 300 counts, isolated width of 2.0 *m/z*, and activation time of 30 ms. The data were viewed and manually analyzed using Xcalibur software (version 2.2, Thermo Scientific).

Using this approach, mucin *O*-glycan structures were characterized based on their molecular mass, monosaccharide composition, MS/MS fragmentation pattern and their retention time on PGC column.

**Staining of mucins bound to PVDF membrane:**

*Dot-blotting of mucins:* the PVDF membrane (Merck Millipore Ltd.) with 0.45 µm pore size and blot paper (Bio-Rad Laboratories) were pretreated with methanol (Sigma-Aldrich Co.) prior to use. Twenty five µg of the purified mucin samples dissolved in 100 µL 4M guanidine hydrochloride (GuHCl) were dot-blotted on PVDF membrane using vacuum. The membrane was air-dried and subjected to Periodic Acid-Schiff (PAS) or AB staining.

*PAS staining:* the membrane was washed with DH_2_O for 2 min and then incubated for 30 min in a freshly prepared solution of 1% periodic acid (v/v) in 3% acetic acid (v/v) at room temperature (RT). The membrane was rinsed twice for 2 min in freshly prepared 0.1% (w/v) sodium-metabisulfite in 1 mM HCl. Schiff’s reagent was added to the membrane and color was allowed to develop for 10 min. The membrane was rinsed twice for 2 min in freshly prepared 0.1% (w/v) sodium-metabisulfite in 1 mM HCl. The membrane was scanned with Bio-rad gel reading instrument and the intensity of the bands was quantified with the ImageJ software.

*AB staining*: AB staining was carried out at pH 2.5 or pH 1.0. The membranes were soaked with 3% acetic acid (pH 2.5, ‘AB 2.5’) or 100 mM HCl (pH 1.0, ‘pH 1.0’). Samples were stained for 1h with 1% Alcian Blue 8GX (Sigma, Lot# MKCH5435) dissolved in 3% (v/v) acetic acid (AB 2.5) or 100 mM HCl (AB 1.0). The same batch of Alcian blue 8GX was used throughout the study. Rinsing of excess stain was carried out with 3% acetic acid (AB 2.5) or 100 mM HCl (AB 1.0). The membrane was destained with methanol three times. The membrane was scanned with Bio-rad gel reading instrument and the intensity of the bands was quantified with the ImageJ software. Signals were normalized for background intensity and differences were expressed in % change (e.g. 20% means 20% stronger staining of mucins than background).

**Histological staining**

Four µM thick sections were used for histology staining after dewaxing. Four Alcian blue pH 1.0 protocols (named #1-4) were tested along with Alcian blue pH 2.5–periodic acid–Schiff (AB-PAS) staining that served as a reference stain (protocol #5). Briefly, sections were soaked for 10 min in 99.5% ethanol and rinsed for 10 min in running tap water. Sections were immersed for 2 min in 0.1 M HCl (# 1-4) or 3% acetic acid (HAc, #5). Staining with Alcian blue was performed with 1% Alcian blue 8GX (Sigma, Lot# MKCH5435) in 0.1M HCl (pH 1.0) for 30 min (#1-4) or in 3% HAc (pH 2.5) for 2.5 h (#5). The same batch of Alcian blue 8GX was used throughout the study. Protocols # 1-4 differed in the destaining process: # 1 slides were rinsed three times with 0.1 M HCl (pH 1.0); # 2 slides were gently blotted with paper; # 3 slides were first rinsed in 0.1 M HCl containing 10% (v/v) MetOh (pH 1.0) three times then rinsed with 0.5 M NaCl/HCl (pH 1.0) three times; #4 slides were rinsed with 0.5 M NaCl/HCl (pH 1.0) three times and gently blotted with paper; #5 slides were immersed in 3% HAc for 1 min and rinsed under running tap water for 9 min. Protocol #1 is a widely recommended staining protocol in AB pH 1.0 kits. Protocol #2 follows Spicers recommendation for avoiding non-specific staining by blotting excess dye with filter paper (Spicer, S.S. 1965). We designed protocol #3 and protocol #4 aiming to reduce unspecific Alcian blue staining. Protocol #3 combines the potential effects of methanol and NaCl to help dissociate weak, unspecific bonds, while protocol #4 combines the effect of NaCl with paper blotting. In # 5 sections were oxidized in 1% periodic acid for 10 min, rinsed under running tap water for 5 min, immersed in 25% Schiff’s reagent (Sigma-Aldrich, St. Louis, MO) and rinsed under running tap water for 5 min. # 5 slides were treated with 0.5% sodium metabisulfite three times for 1 min and washed with tap water. After staining slides from all protocols were dehydrated, and mounted. Scoring of the AB-PAS staining (from 0 to 10) was carried out by visual inspection in a bright-field microscope. To make the images more comparable and representative for the reader we had to adjust the colour of the images slightly. Photos were taken with a Nikon Eclipse 90i microscope with a DS-Vi1 camera.

**References**

Benktander J, Venkatakrishnan V, Padra JT, Sundh H, Sundell K, Murughan AVM, Maynard B, Linden SK. 2019. Effects of size and geographical origin on Atlantic salmon, Salmo salar, mucin O-glycan repertoire. *Mol Cell Proteomics*.

Jin C, Kenny DT, Skoog EC, Padra M, Adamczyk B, Vitizeva V, Thorell A, Venkatakrishnan V, Lindén SK, Karlsson NG. 2017. Structural Diversity of Human Gastric Mucin Glycans. *Molecular & cellular proteomics : MCP*, 16:743-758.

Jin C, Padra JT, Sundell K, Sundh H, Karlsson NG, Linden SK. 2015. Atlantic Salmon Carries a Range of Novel O-Glycan Structures Differentially Localized on Skin and Intestinal Mucins. *Journal of proteome research*, 14:3239-3251.

Padra JT, Sundh H, Sundell K, Venkatakrishnan V, Jin C, Samuelsson T, Karlsson NG, Lindén SK. 2017. Aeromonas salmonicida Growth in Response to Atlantic Salmon Mucins Differs between Epithelial Sites, Is Governed by Sialylated and N-Acetylhexosamine-Containing O-Glycans, and Is Affected by Ca(2). *Infection and immunity*, 85.

Padra M, Adamczyk B, Benktander J, Flahou B, Skoog EC, Padra JT, Smet A, Jin C, Ducatelle R, Samuelsson T*, et al.* 2018. Helicobacter suis binding to carbohydrates on human and porcine gastric mucins and glycolipids occurs via two modes. *Virulence*:1-48.

Padra M, Adamczyk B, Flahou B, Erhardsson M, Chahal G, Smet A, Jin C, Thorell A, Ducatelle R, Haesebrouck F*, et al.* 2019. Helicobacter suis infection alters glycosylation and decreases the pathogen growth inhibiting effect and binding avidity of gastric mucins. *Mucosal immunology*, 12:784-794.

Quintana-Hayashi MP, Mahu M, De Pauw N, Boyen F, Pasmans F, Martel A, Premaratne P, Fernandez HR, Teymournejad O, Vande Maele L*, et al.* 2015. The levels of Brachyspira hyodysenteriae binding to porcine colonic mucins differ between individuals, and binding is increased to mucins from infected pigs with de novo MUC5AC synthesis. *Infection and immunity*, 83:1610-1619.

Sharba S, Venkatakrishnan V, Padra M, Winther M, Gabl M, Sundqvist M, Wang J, Forsman H, Linden SK. 2019. Formyl peptide receptor 2 orchestrates mucosal protection against Citrobacter rodentium infection. *Virulence*, 10:610-624.

Spicer SS. 1965. DIAMINE METHODS FOR DIFFERENTIALING MUCOSUBSTANCES HISTOCHEMICALLY. *The journal of histochemistry and cytochemistry : official journal of the Histochemistry Society*, 13:211-234.

Venkatakrishnan V, Padra JT, Sundh H, Sundell K, Jin C, Langeland M, Carlberg H, Vidakovic A, Lundh T, Karlsson NG*, et al.* 2019. Exploring the Arctic Charr Intestinal Glycome: Evidence of Increased N-Glycolylneuraminic Acid Levels and Changed Host-Pathogen Interactions in Response to Inflammation. *Journal of proteome research*, 18:1760-1773.

Venkatakrishnan V, Quintana-Hayashi MP, Mahu M, Haesebrouck F, Pasmans F, Lindén SK. 2017. Brachyspira hyodysenteriae Infection Regulates Mucin Glycosylation Synthesis Inducing an Increased Expression of Core-2 O-Glycans in Porcine Colon. *Journal of proteome research*, 16:1728-1742.
